# A checklist of xyleborine ambrosia beetles (Coleoptera, Curculionidae, Scolytinae, Xyleborini) on a single fallen chinquapin tree (*Castanopsis inermis*) from Thailand: with a new species and a new country record

**DOI:** 10.3897/BDJ.13.e165806

**Published:** 2025-09-23

**Authors:** Wisut Sittichaya, Sarah M. Smith, Narit Thaochan, Sinlapachai Senarat

**Affiliations:** 1 Agricultural Innovation and Management Division, Faculty of Natural Resources, Prince of Songkla University, 90110, Songkhla, Thailand Agricultural Innovation and Management Division, Faculty of Natural Resources, Prince of Songkla University, 90110 Songkhla Thailand; 2 Michigan State University, East Lansing, Michigan, United States of America Michigan State University, East Lansing Michigan United States of America; 3 Division of Biological Science, Faculty of Science, Prince of Songkla University, Songkhla, Thailand Division of Biological Science, Faculty of Science, Prince of Songkla University Songkhla Thailand

**Keywords:** ambrosia beetles, Xyleborini, new taxon, checklist, Thailand, host plant

## Abstract

**Background:**

The tribe Xyleborini comprises over 1,300 species of ambrosia beetles, all of which form mutualistic relationships with fungi. Several beetle species exhibit strong host preferences, particularly within the plant family Fagaceae for genera such as *Castanopsis*. This study documents the xyleborine beetles associated with *Castanopsis inermis* in Thailand to improve understanding of their host use and diversity.

**New information:**

Twenty species of xyleborine ambrosia beetles were collected from a single fallen *Castanopsis inermis* tree. Amongst them, twelve species were recorded from *C. inermis* for the first time. One species, *Arixyleborus perbrevis* Sittichaya & Smith, sp. nov., is described as new to science, while *Cnestus ater* (Eggers 1923) is newly recorded from Thailand. Some biological observations on xyleborine ambrosia beetles are also presented.

## Introduction

The tribe Xyleborini, within the subfamily Scolytinae (Coleoptera, Curculionidae), is the largest and one of the most ecologically significant lineages of ambrosia beetles, comprising over 1,300 described species globally (Ruzzier et al. 2023). Members of this tribe are characterised by their obligate mutualistic relationships with symbiotic fungi, which they cultivate inside host plant tissues and rely upon for nutrition throughout their development (Hulcr and Stelinski 2017, Biedermann and Vega 2020). These beetles have adapted to a broad range of woody hosts and climates, but they exhibit peak diversity in tropical and subtropical forests (Ruzzier et al. 2023). Due to their wood-boring behaviour and fungal associations, many species in this group are regarded as economically important pests, capable of causing significant damage to forestry and horticultural systems worldwide (Carrillo et al. 2012, Ranger et al. 2016).

Although xyleborine beetles are often considered generalists, accumulating evidence suggests that host specificity plays a more significant role in their ecology and invasion biology than previously assumed. The first comprehensive global dataset on host associations of Xyleborini revealed that, while many species can colonise multiple hosts, they tend to exhibit strong preferences for certain plant families or genera, particularly in their native ranges (Ruzzier et al. 2023). Host plant identity is known to influence not only beetle colonisation success, but also fungal symbiont viability and reproduction (Melet and Biedermann 2024). This complex ecological interplay is increasingly recognised as central to the management of invasive Xyleborini, which are frequently introduced to new regions via international trade of wood and living plants (Poland and Rassati 2019, Gugliuzzo et al. 2021).

More than one hundred and fifty species within Xyleborini have been documented to infest trees in the family Fagaceae, including ecologically and economically important genera, such as *Quercus* Linnaeus, *Lithocarpus* Blume and *Castanopsis* (D. Don) (Ruzzier et al. 2023). In Thailand and surrounding regions, several xyleborine beetles, including newly-described taxa, have been collected from Fagaceae hosts (Eggers 1926, Beaver et al. 2014, Smith et al. 2020). Notably, *Castanopsis inermis* (Lindl.) Benth. & Hook.f., commonly known as chinquapin tree, a native tree species in Southeast Asia, has been understudied in terms of its associated ambrosia beetle fauna. Only thirteen Xyleborini were previously recorded on infested *C. inermis* Ruzzier et al. 2023). Given the ecological importance of *Castanopsis* in Asian montane forests and the increasing detection of xyleborine beetles on Fagaceae hosts (Ruzzier et al. 2023), targeted investigations, such as the present study, are crucial for understanding host-pest dynamics, especially in biodiversity-rich, but data-deficient regions.

Moreover, documenting the Xyleborini community on *Castanopsis inermis* provides a valuable baseline for future ecological monitoring and forest management. *Castanopsis* species are often dominant in canopy trees in subtropical and montane evergreen forests, contributing significantly to forest structure, microclimate regulation and habitat complexity (Ashton and Zhu 2020). In northern Thailand, *Castanopsis* forms a major component of lower montane and hill-evergreen forests, supporting high species richness and structural diversity (Chokchaichamnankit et al. 2008). Baseline data from a single fallen tree can reveal the host-specific beetle assemblage, provide information for long-term monitoring of forest health and provide reference information for biosecurity and conservation planning (Hartshorn et al. 2021, Peng et al. 2022 Ruzzier et al. 2023).

## Materials and methods

**Figure 1.**
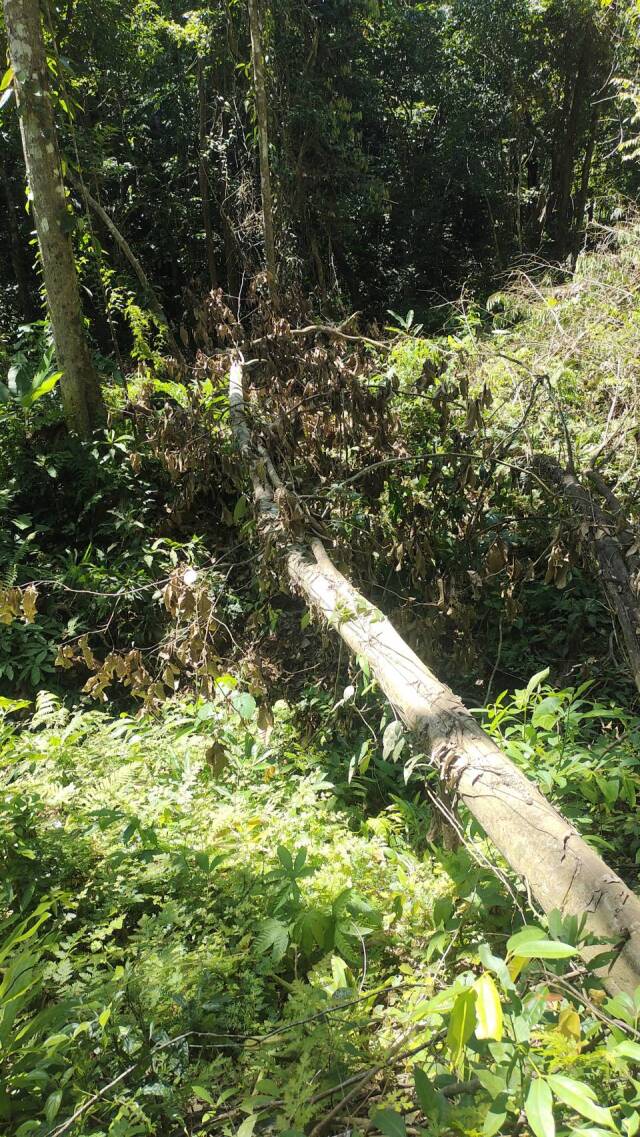
The fallen *Castanopsis inermis* (Fagaceae) where the beetles were collected.

Specimens were extracted from the trunk, main branches, branches and twigs of a single fallen *Castanopsis inermis* tree at the edge of a tropical rainforest, adjacent to Ton Nga Chang Wildlife Sanctuary, Songkhla Province, southern Thailand (Fig. 1). The tree had fallen due to strong winds on an unknown date. The date of falling was estimated from the age of the progeny, which were around three weeks old prior to examination of the wood material. The infested parts of the tree were cut and transported to the laboratory of Agricultural Innovation and Management Division, Faculty of Natural Resources, Prince of Songkla University on 12 August 2024. They were examined using Leica S9D and S8APO microscopes. Bark beetles were extracted from the infested parts of the tree and eggs, larvae, pupae, juveniles and adults were counted. The sex ratio was calculated during the late stage of gallery development using sexually differentiable stages (pupae and adults). Males are recognised by their smaller size in comparison with females. Additional distributional records for the species *Cnestus ater* (Eggers, 1923), a new country record, were collected either by hand or with ethanol-baited traps (eBT.) from different localities, as indicated in the distribution section below. Species were identified using Smith et al. (2020) or by physical comparison with type specimens or photos of type specimens photographed by WS and SMS. Photographs of type specimens of almost all the described species have also been examined by at least one author (SMS). The generic placement of the new taxon was determined in accordance with the outline proposed by Smith et al. (2020). Photographs were taken with a Canon EOS R digital Camera with a Canon MP-E 65 mm Macro Photo Lens (Canon, Tokyo, Japan) and StackShot-Macrorail (Cognisys Inc, Michigan, USA). The photos were then combined with Helicon Focus 8.2.2. (Helicon Soft, Ukraine). The saturation, contrast and clarity of all photos were improved with Adobe Photoshop CS6 (Adobe Systems, California, USA). The terminology for antennal and pronotum types and characters of the new species and new country records follows the terms and classification proposed by Hulcr et al. (2007) and subsequently elaborated by Smith et al. (2020). Length was measured from pronotal anterior margin to the apex of the elytral declivity and width was measured at the widest part of the specimen. Elytral length was measured from the anterior margin to the apex along the elytral medial suture. The pedicel is excluded from the count of number of funicular antennomeres. The specimen collection methodologies were approved by the Animal Care and Use Committee of Prince of Songkla University (Protocol Code:2024-NAT02-007). Unless specified otherwise, detailed information on the hosts, biology and gallery structure of previously described species is available in Browne (1961).

## Taxon treatments

### Arixyleborus perbrevis

Sittichaya & Smith sp. nov.

2E6E2386-FD86-5859-A5F4-360749BA5184

DAB7CF56-1CDA-4878-997B-639D4B98D7C6

#### Materials

- **Type status:** Holotype. **Occurrence:** recordedBy: W. Sittichaya; individualCount: 1; sex: female; lifeStage: adult; disposition: Naturhistorisches Museum Wien; occurrenceID: DD8C905C-B8BB-5E51-9052-1E6A0D802F8C; **Taxon:** kingdom: Animalia; phylum: Arthropoda; class: Insecta; order: Coleoptera; family: Curculionidae; genus: Arixyleborus; specificEpithet: *perbrevis*; taxonRank: species; nomenclaturalStatus: sp.nov; **Location:** locationID: Hat Yai District; continent: Asia; country: Thailand; stateProvince: Songkhla Province; county: Thailand; verbatimElevation: 120 m; verbatimLatitude: 6°55'20.8"; verbatimLongitude: 100°14'48.8"; **Event:** samplingProtocol: ex. small branch of fallen *Castanopsis inermis* (Fagaceae); eventDate: 12.viii.2024; year: 2024; habitat: tropical rainforest

#### Description

**Figure 2.**
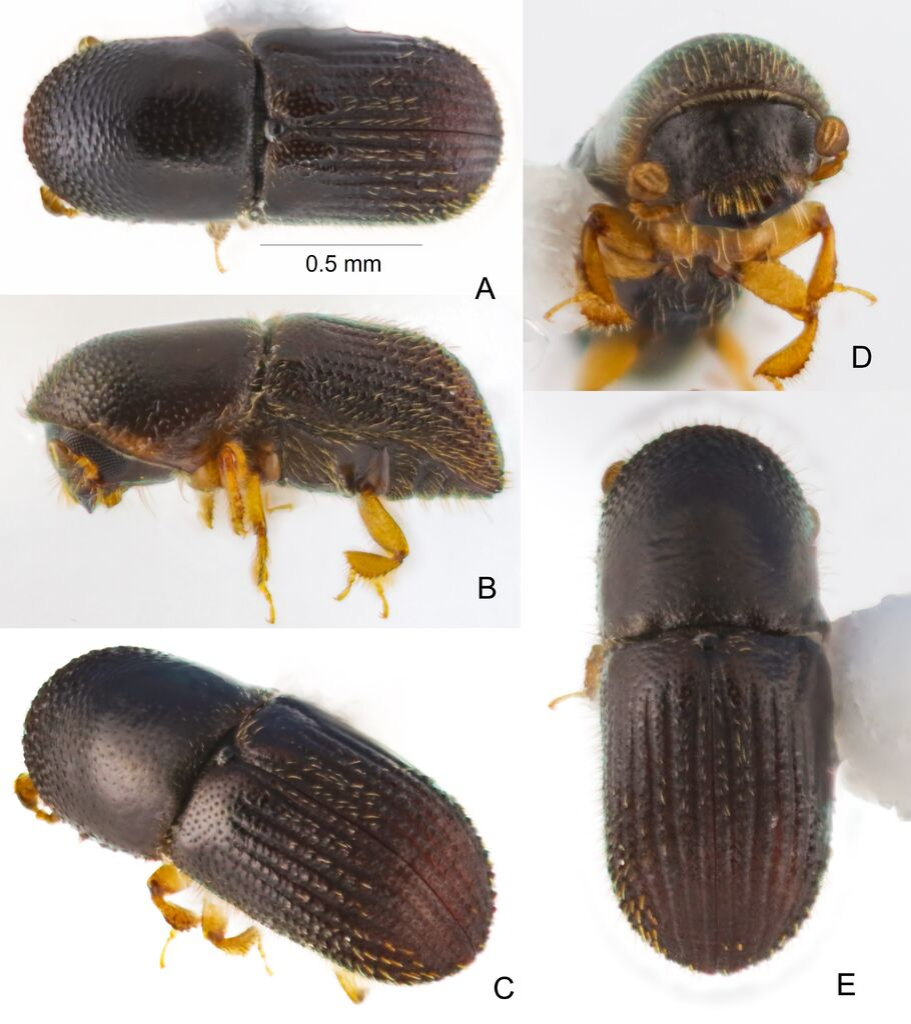
*Arixyleborus perbrevis* Sittichaya & Smith sp. nov., holotype. **A** dorsal view; **B** lateral view; **C** postero-lateral view; **D** frons; **E** posterior-dorsal view showing elytral declivity.

(Fig. 2) Female. 1.56 mm long (n = 1), 0.6 mm wide, 2.60× as long as wide. Body dark brown; appendages paler. **Head.** Epistoma entire, sinuate, with a row of sparse, short, hair-like, greenish-gold setae. Frons feebly convex from epistoma to dorsal margin of eyes; surface alutaceous and subshiny. Lower part of frons without a distinct medial line, upper part with a short, small, subshiny medial line. Lower half of frons granulate-punctate; granules moderate in size and moderately sparse. Upper half of frons punctate without granules; punctures small and shallow, each with a greenish-gold seta (mostly abraded). Eyes emarginate above the level of antennal insertion; upper portion slightly smaller than the lower. Submentum deeply impressed, very narrow and triangular. Antennal scape thick, slightly longer than club (6/5.5). Pedicel as long as funicle. Antennal funicle with 4 antennomeres; antennomere 1 shorter than pedicel. Club as long as wide, type 1, obliquely truncate; club antennomere 1 occupies approximately the basal half, concave on anterior side, with a sharp marginal costa; antennomere 2 narrow, pubescent, without sclerotised part, antennomere 2 not visible on posterior face, visible only on anterior face.

**Pronotum**: 0.7 mm long, 0.6 mm wide, 1.17× as long as wide. In dorsal view type 7, long and rounded anteriorly, sides parallel in posterior 2/3; anterior margin without serrations. In lateral view, type 8 disc much longer than anterior slope, summit low. Anterior slope finely asperate, asperities low, closely spaced, with very fine, short hair-like setae. Disc alutaceous, shiny, with fine, moderately dense punctures, some short, suberect setae present on lateral and posterior margins. Lateral margins obliquely costate. Posterior margin weakly concave. **Elytra.** 0.81 mm long, 0.60 mm wide, 1.35× longer than wide, 1.16× longer than pronotum. Scutellar shield moderately sized, subrectangular, slightly elevated above adjacent part of elytra. Elytral bases weakly bisinuate, edge oblique, elytral area adjacent to scutellar shield weakly impressed, humeral angles rounded. Sides parallel from base to apical 1/2 of declivity, then rounded to apex. Disc 1.3× as long as declivity, anterior 1/3 shiny, posterior 2/3 dull; striae and interstriae flat in anterior 1/4, densely and shallowly punctured, each puncture bearing a very fine, hair-like seta, very short on striae, longer on interstriae (almost abraded in holotype); posterior 3/4 of disc with striae increasingly deeply impressed towards apex; interstriae raised, each with a single row of irregularly placed granules increasing in size posteriorly to declivital summit; striae 2‒3× narrower than interstriae; vestiture with minute strial hair-like setae and interstriae with 2 or 3 irregular rows of longer, stouter setae (almost abraded in holotype). Declivity abruptly rounded, very steep; declivital face distinctly convex, strongly shagreened, dull; striae feebly impressed, impunctate, wider than on disc and slightly wider than interstriae; interstriae feebly elevated, distinctly lower than that on disc, without granules, decreasing in size towards apex; interstriae 1‒4 reaching apex of declivity, bearing flattened, stout, bristle-like setae (almost abraded in holotype); outer interstriae abbreviated, not reaching apex, with long, hair-like setae as on disc. Posterolateral margin of elytra costate, denticulate, to interstriae 7. **Legs.** Protibiae slender, widest in apical 1/4; posterior face inflated, strongly granulate; outer margin with five distinctly separated, socketed denticles in apical half. Meso- and metatibiae flattened, outer margin evenly rounded with eight socketed denticles; posterior face unarmed.

#### Diagnosis

1.56 mm long (n = 1), 0.6 mm wide, 2.60× as long as wide. This species is distinguished by combination of the following characters: pronotum type 7 in dorsal view, elongate and rounded anteriorly; type 8 in lateral view with a low summit and a distinctly longer disc than the anterior slope; elytral disc 1.3× as long as the declivity; elytral striae and interstriae flat in the anterior quarter; elytral interstriae raised and irregularly granulate apically, with granules increasing in size posteriorly; declivity abruptly steep, rounded, strongly shagreened, convex; declivital interstriae elevated lower than that on disc, without granules, striae impunctate without vestiture.

This species resembles *A. rugosipes*, but can be distinguished as follows (with *A. perbrevis* characters given first): smaller body size, 1.56 mm long vs. larger, 1.7–2.0 mm; body shorter, 2.60× as long as wide vs. more elongate, 2.83–3.33× as long as wide; declivity abruptly rounded and very steep vs. gradually descending.

The generic placement of *Arixyleborus perbrevis* was determined in accordance with the outline proposed by Smith et al. (2020).

#### Etymology

Latin adjective, *perbrevis* = short, referred to its short elytra compared to related species.

#### Distribution

THAILAND: Songkhla Province (recorded only from holotype).

#### Biology

This species was found breeding in a small branch of *Castanopsis inermis*.

## Checklists

### A checklist of xyleborine ambrosia beetles on a single fallen chinquapin tree (Castanopsis inermis) from Thailand

#### Amasa macarthorum

Sittichaya & Smith, 2022

3C7E06C9-33F8-5A8D-920B-57AE1FD85BD7

##### Materials

- **Type status:** Other material. **Occurrence:** individualCount: 5; sex: female; disposition: Wisut Sittichaya Private Collection; occurrenceID: 2713AFF2-8E2A-5E58-B412-5AE1F87A30F4; **Taxon:** kingdom: Animalia; phylum: Arthropoda; class: Insecta; order: Coleoptera; family: Curculionidae; genus: Amasa; specificEpithet: macarthorum; taxonRank: species; scientificNameAuthorship: Sittichaya & Smith, 2022; taxonomicStatus: valid; **Location:** country: Thailand; locality: Songkhla Province, Hat Yai District; verbatimElevation: 120 m; verbatimCoordinates: 6°55'20.8"N 100°14'48.8"E; **Identification:** identifiedBy: Sittichaya & Smith; **Event:** eventDate: 12.viii.2024; habitat: tropical rainforest; **Record Level:** type: Non type

##### Distribution

Thailand: Chaiyaphum, Nakhon Sri Thammarat, Songkhla.

##### Notes

**Biology**: The hosts and biology of *A. macarthorum* was previously unknown. This species breeds in small branches (2–4 cm in diameter) of *C. inermis* (newly-recorded host). Eggs were deposited in 2–3 clusters per gallery, each cluster contained 4–6 eggs. Developmental stages of progeny varied amongst individuals within each gallery. A total of 12–26 progeny were recorded per gallery (n = 5), with a strongly female-biased sex ratio of 16:1 (n = 2).

#### Amasa versicolor

(Sampson, 1921)

4BC7343C-B603-5467-80AD-45530920101E

##### Materials

- **Type status:** Other material. **Occurrence:** individualCount: 1; sex: female; disposition: Wisut Sittichaya Private Collection; occurrenceID: DAED4CAF-794F-54D7-957F-52097495D463; **Taxon:** kingdom: Animalia; phylum: Arthropoda; class:  Insecta; order: Coleoptera; family: Curculionidae; genus: Amasa; specificEpithet: versicolor; taxonRank: species; scientificNameAuthorship: (Sampson, 1921); taxonomicStatus: valid; **Location:** country: Thailand; locality: Songkhla Province, Hat Yai District; verbatimElevation: 120 m; verbatimCoordinates: 6°55'20.8"N 100°14'48.8"E; **Identification:** identifiedBy: Sittichaya & Smith; **Event:** eventDate: 12.viii.2024; habitat: tropical rainforest; **Record Level:** type: Non type

##### Distribution

Thailand: Nakhon Sri Thammarat, Songkhla Province.

##### Notes

**Biology**: This species breeds in 3 cm branch of *C. inermis* (newly-recorded host); eggs were deposited in two clusters of nine and six eggs, respectively (n = 1).

#### Arixyleborus leprosulus

Schedl, 1953

0C10092D-CD81-54FC-9E94-0FC4BEFEF055

##### Materials

- **Type status:** Other material. **Occurrence:** individualCount: 1; sex: female; occurrenceID: F88030E0-6AB8-5137-85A6-50A769595E1A; **Taxon:** kingdom: Animalia; phylum: Arthropoda; class: Insecta; order: Coleoptera; family: Curculionidae; genus: Arixyleborus; specificEpithet: leprosulus; taxonRank: species; scientificNameAuthorship: Schedl, 1953; taxonomicStatus: valid; **Location:** country: Thailand; locality: Songkhla Province, Hat Yai District; verbatimElevation: 120 m; verbatimCoordinates: 6°55'20.8"N 100°14'48.8"E; **Identification:** identifiedBy: Sittichaya & Smith; **Event:** eventDate: 12.viii.2024; habitat: tropical rainforest; **Record Level:** type: Non type

##### Distribution

Thailand: Chumphon, Narathiwat, Songkhla, Surat Thani.

##### Notes

**Biology**: This species was found in 3 cm branch of *C. inermis*.

#### Arixyleborus malayensis

(Schedl, 1954)

47E16B16-C3E0-5790-B541-856BDCECE4EB

##### Materials

- **Type status:** Other material. **Occurrence:** individualCount: 1; sex: female; disposition: Wisut Sittichaya Private Collection; occurrenceID: 0C35ACE2-D111-58E2-AEFB-20F0A508AD79; **Taxon:** kingdom: Animalia; phylum: Arthropoda; class:  Insecta; order: Coleoptera; family: Curculionidae; genus: Arixyleborus; specificEpithet: malayensis; taxonRank: species; scientificNameAuthorship: (Schedl, 1954); taxonomicStatus: valid; **Location:** country: Thailand; locality: Songkhla Province, Hat Yai District; verbatimElevation: 120 m; verbatimCoordinates: 6°55'20.8"N 100°14'48.8"E; **Identification:** identifiedBy: Sittichaya & Smith; **Event:** eventDate: 12.viii.2024; habitat: tropical rainforest; **Record Level:** type: Non type

##### Distribution

Thailand: Chiang Mai, Chumphon, Kanchanaburi, Mae Hong Son, Nakhon Nayok, Nan, Rayong, Songkhla, Surat Thani.

##### Notes

**Biology**: This species was found in 2.5 cm branch of *C. inermis* (newly-recorded host).

#### Arixyleborus rugosipes

Hopkins, 1915

5FE2BCCD-ED2A-5812-97A4-83D3E5C76E16

##### Materials

- **Type status:** Other material. **Occurrence:** individualCount: 1; sex: female; disposition: Wisut Sittichaya Private Collection; occurrenceID: 014CE1CA-6B27-5965-A034-B11914723F39; **Taxon:** kingdom: Animalia; phylum: Arthropoda; class:  Insecta; order: Coleoptera; family: Curculionidae; genus: Arixyleborus; specificEpithet: rugosipes; taxonRank: species; scientificNameAuthorship: Hopkins, 1915; taxonomicStatus: valid; **Location:** country: Thailand; locality: Songkhla Province, Hat Yai District; verbatimElevation: 120 m; verbatimCoordinates: 6°55'20.8"N 100°14'48.8"E; **Identification:** identifiedBy: Sittichaya & Smith; **Event:** eventDate: 12.viii.2024; habitat: tropical rainforest; **Record Level:** type: Non type

##### Distribution

Thailand: Chumphon, Nakhon Sri Thammarat, Phangnga, Songkhla, SuratThani, Trang.

##### Notes

**Biology**: This species was found in 1.5 cm branch of *C. inermis* (newly-recorded host).

#### Arixyleborus suturalis

(Eggers, 1936)

7EF40B43-AE29-5006-B345-6341FFC72F12

##### Materials

- **Type status:** Other material. **Occurrence:** individualCount: 1; sex: female; disposition: Wisut Sittichaya Private Collection; occurrenceID: 0AC972E3-0E4F-53DD-987D-38D4AE92AD87; **Taxon:** kingdom: Animalia; phylum: Arthropoda; class:  Insecta; order: Coleoptera; family: Curculionidae; genus: Arixyleborus; specificEpithet: rugosipes; taxonRank: species; scientificNameAuthorship: Hopkins, 1915; taxonomicStatus: valid; **Location:** country: Thailand; locality: Songkhla Province, Hat Yai District; verbatimElevation: 120 m; verbatimCoordinates: 6°55'20.8"N 100°14'48.8"E; **Identification:** identifiedBy: Sittichaya & Smith; **Event:** eventDate: 12.viii.2024; habitat: tropical rainforest; **Record Level:** type: Non type

##### Distribution

Thailand: Chiang Mai, Nakhon Sri Thammarat, Songkhla.

##### Notes

**Biology**: This species was found in 2 cm branch of *C. inermis*.

#### Cnestus ater

(Eggers, 1923)

7AD697AF-E60D-5225-BA06-E68184AEC216

##### Materials

- **Type status:** Other material. **Occurrence:** individualCount: 1; sex: female; disposition: Wisut Sittichaya Private Collection; occurrenceID: 9337EA58-BC04-502D-BF06-DB4E4F6943FE; **Taxon:** kingdom: Animalia; phylum: Arthropoda; class: Insecta; order: Coleoptera; family: Curculionidae; genus: Cnestus; specificEpithet: ater; taxonRank: species; scientificNameAuthorship: (Eggers, 1923); taxonomicStatus: valid; **Location:** country: Thailand; locality: Songkhla Province, Hat Yai District; verbatimElevation: 120 m; verbatimCoordinates: 6°55'20.8"N 100°14'48.8"E; **Identification:** identifiedBy: Sittichaya & Smith; **Event:** samplingProtocol: ex. 4.5 cm. diameter branch of fallen C. inermis; eventDate: 12.viii.2025; habitat: tropical rainforest; **Record Level:** type: Non type
- **Type status:** Other material. **Occurrence:** individualCount: 6; sex: female; disposition: Wisut Sittichaya Private Collection; occurrenceID: 1E8BAD82-2760-5B01-A55B-BD288A03C6CE; **Location:** locality: Trat Province, Koh Kood Island; verbatimElevation: 65; verbatimCoordinates: 11°38'43.8"N 102°33'43.6"E; **Identification:** identifiedBy: Sittichaya; **Event:** samplingProtocol: ex. small branches of Saraca asoca; eventDate: 01.ii.23; habitat: tropical rainforest; **Record Level:** type: Non type
- **Type status:** Other material. **Occurrence:** individualCount: 5; sex: female; disposition: Wisut Sittichaya Private Collection; occurrenceID: C0860BDA-38AB-56A0-A280-91D735BCA91B; **Location:** locality: Sa Kaeo Province; verbatimElevation: 380; verbatimCoordinates: 14°02'21.8"N 102°16'15.5"E; **Identification:** identifiedBy: Sittichaya; **Event:** samplingProtocol: ethanol baited trap (eBT.); eventDate: 01-31.i.2021; habitat: tropical rainforest; **Record Level:** type: Non type
- **Type status:** Other material. **Occurrence:** individualCount: 1; sex: female; disposition: Wisut Sittichaya Private Collection; occurrenceID: 8C49A0D9-8914-57B3-B454-7FC1A4E534E1; **Location:** locality: Ranong Province, Klongnakha wildlife sanctuary; verbatimCoordinates: 9°28'03.9"N 98°32'06.7"E; verbatimLatitude: 215; **Identification:** identifiedBy: Sittichaya; **Event:** samplingProtocol: ethanol baited trap (eBT.); eventDate: 01.vi.2014; habitat: tropical rainforest; **Record Level:** type: Non type
- **Type status:** Other material. **Occurrence:** individualCount: 1; sex: female; disposition: Wisut Sittichaya Private Collection; occurrenceID: C6682410-F690-5E04-A611-648A6E9F5C4E; **Location:** locality: Trang Province, Khao Bhantad wildlife sanctuary; **Identification:** identifiedBy: Sittichaya; **Event:** samplingProtocol: ethanol baited trap (eBT.); eventDate: 01.i.2014; habitat: tropical rainforest; **Record Level:** type: Non type
- **Type status:** Other material. **Occurrence:** individualCount: 1; sex: female; occurrenceID: D33CD3FF-F70C-5030-AFF8-7693B9C36445; **Location:** locality: Songkhla Province, Ton Ngachang wildlife sanctuary; verbatimElevation: 290; verbatimCoordinates: 6°58'48.9"N 100°09'10.5"E; **Identification:** identifiedBy: Sittichaya; **Event:** samplingProtocol: ethanol baited trap (eBT.); eventDate: 01.x.2015; habitat: tropical rainforest; **Record Level:** type: Non type
- **Type status:** Other material. **Occurrence:** individualCount: 3; sex: female; disposition: Wisut Sittichaya Private Collection; occurrenceID: 5349638C-84E2-5354-8778-204C6EEFCE25; **Location:** locality: Narathiwat Province, Hala-Bala wildlife sanctuary; verbatimElevation: 96; verbatimCoordinates: 5°47'42.2"N 101°49'35.4"E; **Identification:** identifiedBy: Sittichaya; **Event:** samplingProtocol: ethanol baited trap (eBT.); eventDate: 01.x.2015 (2), 01.vii.2014 (1); habitat: lowland tropical rainforest; **Record Level:** type: Non type

##### Distribution

This species is recorded in Thailand for the first time. Thailand: Narathiwat, Ranong, Sa Kaeo, Trang, Trat.

##### Notes

This species was found breeding in a 4.5 cm diameter branch of *C. inermis* (newly-recorded host), small branches of *Saraca asoca* (Fabaceae) (newly-recorded host) and 3–8 progeny per gallery were observed.

##### Diagnosis

**Figure 3.**
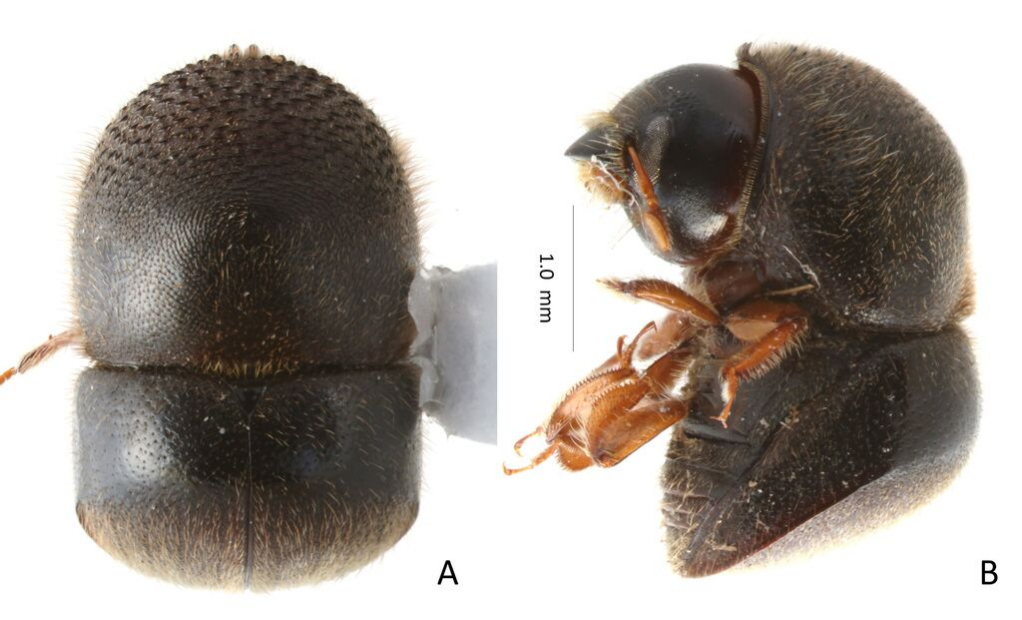
*Cnestus ater* (Eggers, 1923). **A** dorsal view; **B** lateral view.

(Fig. 3) Large body size. 3.48−3.80 mm long (mean = 3.68 mm; n = 6); 1.55−1.65 times as long as wide. This species is distinguished by a short and stout body form, elytra shorter than pronotum, elytral disc very short, less than 1/2 of elytral length, declivity obliquely truncate, abruptly descending from disc, posterior margin of pronotum with mycangial tuft. Scutellar shield of normal size; elytral disc 4–5 scutellar shield lengths; declivital summit rounded, never costate.

#### Cyclorhipidion perpilosellum

(Schedl, 1935)

EDEF6894-6E00-5F09-912F-31708E0D25B9

##### Materials

- **Type status:** Other material. **Occurrence:** individualCount: 10; sex: female; disposition: Wisut Sittichaya Private Collection; occurrenceID: 015D3A7B-ECFC-5F10-85AA-A7B92575FEBD; **Taxon:** kingdom: Animalia; phylum: Arthropoda; class:  Insecta; order: Coleoptera; family: Curculionidae; genus: Cyclorhipidion; specificEpithet: perpilosellum; taxonRank: species; scientificNameAuthorship: (Schedl, 1935); taxonomicStatus: valid; **Location:** country: Thailand; locality: Songkhla Province, Hat Yai District; verbatimElevation: 120 m; verbatimCoordinates: 6°55'20.8"N 100°14'48.8"E; **Identification:** identifiedBy: Sittichaya & Smith; **Event:** eventDate: 12.viii.2024; habitat: tropical rainforest; **Record Level:** type: Non type

##### Distribution

Thailand: Chaiyaphum, Chiang Mai, Nakhon Ratchasima, Nakhon Sri Thammarat, Songkhla, Trang.

##### Notes

This species breeds throughout the *C. inermis* tree, including the trunk, main branches and secondary branches (3–6 cm in diameter). Eggs are laid in 2–3 clusters per gallery, each containing 6–8 eggs. Progeny within a gallery often exhibit a wide range of developmental stages. A total of 15–30 progeny were recorded per gallery (n = 10).

#### Debus quadrispinus

(Motschulsky, 1863)

E0EB01CC-9EE3-585D-A550-8F6847EC80F1

##### Materials

- **Type status:** Other material. **Occurrence:** individualCount: 1; sex: female; disposition: Wisut Sittichaya Private Collection; occurrenceID: A80D7593-E661-5B68-A5C0-9850375E3910; **Taxon:** kingdom: Animalia; phylum: Arthropoda; class:  Insecta; order: Coleoptera; family: Curculionidae; genus: Debus; specificEpithet: quadrispinus; taxonRank: species; scientificNameAuthorship: (Motschulsky, 1863); taxonomicStatus: valid; **Location:** country: Thailand; locality: Songkhla Province, Hat Yai District; verbatimElevation: 120 m; verbatimCoordinates: 6°55'20.8"N 100°14'48.8"E; **Identification:** identifiedBy: Sittichaya & Smith; **Event:** eventDate: 12.viii.2024; habitat: tropical rainforest; **Record Level:** type: Non type

##### Distribution

Thailand: Chiang Mai, Nakhon Sri Thammarat, Songkhla, Surat Thani, Tak, Trang.

##### Notes

This species was found in a 6 cm diameter branch of *C. inermis* (n = 1) (newly-recorded host).

#### Eccoptopterus limbus

Sampson, 1911

23F15108-4691-5DDE-8932-58AFD598EFE5

##### Materials

- **Type status:** Other material. **Occurrence:** individualCount: 1; sex: female; disposition: Wisut Sittichaya Private Collection; occurrenceID: 5191C29E-1A96-5522-8013-10A05F25BE68; **Taxon:** kingdom: Animalia; phylum: Arthropoda; class:  Insecta; order: Coleoptera; family: Curculionidae; genus: Eccoptopterus; specificEpithet: limbus; taxonRank: species; scientificNameAuthorship: Sampson, 1911; taxonomicStatus: valid; **Location:** country: Thailand; locality: Songkhla Province, Hat Yai District; verbatimElevation: 120 m; verbatimCoordinates: 6°55'20.8"N 100°14'48.8"E; **Identification:** identifiedBy: Sittichaya & Smith; **Event:** eventDate: 12.viii.2024; habitat: tropical rainforest; **Record Level:** type: Non type

##### Distribution

Thailand: Chiang Mai, Loei, Songkhla, Surat Thani, Ubon Ratchathani.

##### Notes

This species was found in 2 cm branch of *C. inermis*.

#### Euwallacea similis

(Ferrari, 1867)

2792E732-2EBE-5DF5-BD75-DB88AEFD0E32

##### Materials

- **Type status:** Other material. **Occurrence:** individualCount: 1; sex: female; disposition: Wisut Sittichaya Private Collection; occurrenceID: 708B0924-1CD2-5A4D-946D-F5E6A8DA7E66; **Taxon:** kingdom: Animalia; phylum: Arthropoda; class:  Insecta; order: Coleoptera; family: Curculionidae; genus: Euwallacea; specificEpithet: similis; taxonRank: species; scientificNameAuthorship: (Ferrari, 1867); taxonomicStatus: valid; **Location:** country: Thailand; locality: Songkhla Province, Hat Yai District; verbatimElevation: 120 m; verbatimCoordinates: 6°55'20.8"N 100°14'48.8"E; **Identification:** identifiedBy: Sittichaya & Smith; **Event:** eventDate: 12.viii.2024; habitat: tropical rainforest; **Record Level:** type: Non type

##### Distribution

Thailand: More than 40 provinces in all regions of the country (Sittichaya W., personal surveys).

##### Notes

This species was found in 3 cm branch of *C. inermis* (newly-recorded host).

#### Hadrodemius globus

(Blandford, 1896)

8F018D21-F5BF-54AF-869D-C112D00F9F90

##### Materials

- **Type status:** Other material. **Occurrence:** individualCount: 2; sex: female; disposition: Wisut Sittichaya Private Collection; occurrenceID: 46690AAF-7658-55FF-A089-D879B20B91BF; **Taxon:** kingdom: Animalia; phylum: Arthropoda; class:  Insecta; order: Coleoptera; family: Curculionidae; genus: Hadrodemius; specificEpithet: globus; taxonRank: species; scientificNameAuthorship: (Blandford, 1896); taxonomicStatus: valid; **Location:** country: Thailand; locality: Songkhla Province, Hat Yai District; verbatimElevation: 120 m; verbatimCoordinates: 6°55'20.8"N 100°14'48.8"E; **Identification:** identifiedBy: Sittichaya & Smith; **Event:** eventDate: 12.viii.2024; habitat: tropical rainforest; **Record Level:** type: Non type

##### Distribution

Thailand: Chiang Mai, Nakhon Nayok, Nakhon Ratchasima, Nakhon Sri Thammarat, Sa Kaeo, Songkhla.

##### Notes

This species breeds in 3–6 cm branches of *C. inermis* (newly-recorded host). Two galleries had 6 and 25 progeny, with a sex ratio (female:male) 5:1 and 23:2, respectively.

#### Leptoxyleborus sordicauda

(Motschulsky, 1863)

E0A764D5-1E02-50E3-8B88-67F67E10E6B9

##### Materials

- **Type status:** Other material. **Occurrence:** individualCount: 1; sex: female; disposition: Wisut Sittichaya Private Collection; occurrenceID: C423C371-F3D5-5E9E-B6DE-50427652CDE1; **Taxon:** kingdom: Animalia; phylum: Arthropoda; class:  Insecta; order: Coleoptera; family: Curculionidae; genus: Leptoxyleborus; specificEpithet: sordicauda; taxonRank: species; scientificNameAuthorship: (Motschulsky, 1863); taxonomicStatus: valid; **Location:** country: Thailand; locality: Songkhla Province, Hat Yai District; verbatimElevation: 120 m; verbatimCoordinates: 6°55'20.8"N 100°14'48.8"E; **Identification:** identifiedBy: Sittichaya & Smith; **Event:** eventDate: 12.viii.2024; habitat: tropical rainforest; **Record Level:** type: Non type

##### Distribution

Thailand: Chanthaburi, Chiang Mai, Kanchanaburi, Nakhon Sri Thammarat, Sa Kaeo, Songkhla, Surat Thani, Trang.

##### Notes

This species was found in 6 cm diameter branch of *C. inermis* (n = 1) (newly-recorded host).

#### Microperus perparvus

(Sampson, 1922b)

CD251048-5A22-56B1-8B1C-751239A95D75

##### Materials

- **Type status:** Other material. **Occurrence:** individualCount: 1; sex: female; disposition: Wisut Sittichaya Private Collection; occurrenceID: C65217B5-B6E2-5365-A3A5-29A30779DA34; **Taxon:** kingdom: Animalia; phylum: Arthropoda; class:  Insecta; order: Coleoptera; family: Curculionidae; genus: Microperus; specificEpithet: perparvus; taxonRank: species; scientificNameAuthorship: (Sampson, 1922b); taxonomicStatus: valid; **Location:** country: Thailand; locality: Songkhla Province, Hat Yai District; verbatimElevation: 120m; verbatimCoordinates: 6°55'20.8"N 100°14'48.8"E; **Identification:** identifiedBy: Sittichaya & Smith; **Event:** eventDate: 12.viii.2024; habitat: tropical rainforest; **Record Level:** type: Non type

##### Distribution

Thailand: Chiang Mai, Chaiyaphum, Mae Hong Son, Nakhon Nayok, Nakhon Sri Thammarat, Nan, Phetchabun, Phitsanulok, Loei, Songkhla, Surat Thani.

##### Notes

**Biology**: This species was found in a 6 cm diameter branch of *C. inermis* (n = 1).

#### Tricosa cattienensis

Cognato, Smith & Beaver, 2020

0E7D5213-9820-5B69-AAED-F40D41CD6B74

##### Materials

- **Type status:** Other material. **Occurrence:** individualCount: 1; sex: female; disposition: Wisut Sittichaya Private Collection; occurrenceID: 55FC4DF0-3402-5682-8FB8-90B69C3C9E2E; **Taxon:** kingdom: Animalia; phylum: Arthropoda; class:  Insecta; order: Coleoptera; family: Curculionidae; genus: Tricosa; specificEpithet: cattienensis; taxonRank: species; scientificNameAuthorship: Cognato, Smith & Beaver, 2020; taxonomicStatus: valid; **Location:** country: Thailand; locality: Songkhla Province, Hat Yai District; verbatimElevation: 120 m; verbatimCoordinates: 6°55'20.8"N 100°14'48.8"E; **Identification:** identifiedBy: Sittichaya & Smith; **Event:** eventDate: 12.viii.2024; habitat: tropical rainforest; **Record Level:** type: Non type

##### Distribution

Thailand: Chaiyaphum, Chiang Mai, Phetchaburi, Songkhla, Surat Thani.

##### Notes

This species was found in 5 cm diameter branch of *C. inermis* (n = 1) (newly-recorded host). Additional hosts are given in Cognato et al. (2020).

#### Webbia diversicauda

Browne, 1972

FC14B769-93AD-5FF6-A7BE-937C63B034A5

##### Materials

- **Type status:** Other material. **Occurrence:** individualCount: 20; sex: female; disposition: Wisut Sittichaya Private Collection; occurrenceID: 60E00F5C-1824-502D-B196-FFCD2516ED4D; **Taxon:** kingdom: Animalia; phylum: Arthropoda; class:  Insecta; order: Coleoptera; family: Curculionidae; genus: Webbia; specificEpithet: diversicauda; taxonRank: species; scientificNameAuthorship: Browne, 1972; taxonomicStatus: valid; **Location:** country: Thailand; locality: Songkhla Province, Hat Yai District; verbatimElevation: 120 m; verbatimCoordinates: 6°55'20.8"N 100°14'48.8"E; **Identification:** identifiedBy: Sittichaya & Smith; **Event:** eventDate: 12.viii.2024; habitat: tropical rainforest; **Record Level:** type: Non type

##### Distribution

Thailand: Chaiyaphum, S Nakhon Ratchasima, Narathiwat, Songkhla.

##### Notes

The hosts and biology of this species was previously unknown. This species breeds in 1.2-2.5 cm branches of *C. inermis* (new host recorded). Each gallery (n = 15) contained 2–3 egg clusters. Progeny numbers ranged from 25–38 at various developmental stages. The recorded sex ratio was highly female-biased, ranging from 24:1 to 38:1 (n = 5). In comparatively dry branches of *C. innermis*, brood size was significantly reduced, with only 5–6 progeny (n = 5) and a single egg cluster per gallery.

#### Xyleborus affinis

Eichhoff, 1868

9173A8C3-7877-59AB-8305-EF02E7F40313

##### Materials

- **Type status:** Other material. **Occurrence:** individualCount: 15; sex: female; disposition: Wisut Sittichaya Private Collection; occurrenceID: 43C68396-E966-5ED0-9EF4-03ED66DA3B76; **Taxon:** kingdom: Animalia; phylum: Arthropoda; class:  Insecta; order: Coleoptera; family: Curculionidae; genus: Xyleborus; specificEpithet: affinis; taxonRank: species; scientificNameAuthorship: Eichhoff, 1868; taxonomicStatus: valid; **Location:** country: Thailand; locality: Songkhla Province, Hat Yai District; verbatimElevation: 120 m; verbatimCoordinates: 6°55'20.8"N 100°14'48.8"E; **Identification:** identifiedBy: Sittichaya & Smith; **Event:** eventDate: 12.viii.2024; habitat: tropical rainforest; **Record Level:** type: Non type

##### Distribution

Thailand: More than 40 provinces in all regions of the country (WS, personal surveys).

##### Notes

This species breeds in all parts of the *C. inermis* tree (newly-recorded host), but is more abundant in larger sections of the trunk and main branches. Recorded progenies: 10–15 progeny with different ages, 3–4 clusters per brood (n = 15), sex ratio of 10–15:1 (n = 3).

#### Xyleborus perforans

(Wollaston, 1857)

D8308017-06F3-5B28-BB25-2848644491F2

##### Materials

- **Type status:** Other material. **Occurrence:** individualCount: 20; sex: female; disposition: Wisut Sittichaya Private Collection; occurrenceID: 4561C9C8-4B93-5C44-9504-23563BF23D5C; **Taxon:** kingdom: Animalia; phylum: Arthropoda; class:  Insecta; order: Coleoptera; family: Curculionidae; genus: Xyleborus; specificEpithet: perforans; taxonRank: species; scientificNameAuthorship: (Wollaston, 1857); taxonomicStatus: valid; **Location:** country: Thailand; locality: Songkhla Province, Hat Yai District; verbatimElevation: 120 m; verbatimCoordinates: 6°55'20.8"N 100°14'48.8"E; **Identification:** identifiedBy: Sittichaya & Smith; **Event:** eventDate: 12.viii.2024; habitat: tropical rainforest; **Record Level:** type: Non type

##### Distribution

S. Thailand: More than 40 provinces in all regions of the country (WS, personal surveys).

##### Notes

This species breeds in all parts of the *C. inermis* tree (newly-recorded host), including the trunk, main branches to secondary branches (3–12 cm diameter), except twigs, with more galleries on trunk and main branches. Breeding occurs either in the thick bark, the wood or both. Galleries typically begin as long tunnels in the bark before penetrating deeply into the wood – a pattern also observed in durian (*Durio zibethinus* L.; Bombacaceae) and rubber trees (*Hevea brasiliensis* (Willd. ex A.Juss.) Müll.Arg.; Euphorbiaceae) (WS, personal observation); the gallery is radially branched (2–3 branches) or unbranched. Eggs are laid in both the bark and the wood portions of the gallery. Each gallery (n = 10) consists of 3–5 egg clusters, each containing 3–8 eggs. A total of 10–30 progeny of varying developmental stages were observed per brood, with a recorded sex ratio of 9–18 females per male (n = 5).

#### Xylosandrus mancus

(Blandford, 1898)

0131E5CC-F856-5912-9230-47975A5CC83D

##### Materials

- **Type status:** Other material. **Occurrence:** individualCount: 5; sex: female; disposition: Wisut Sittichaya Private Collection; occurrenceID: 98FED33C-9CE5-5F00-9F9B-039485D2EE6C; **Taxon:** kingdom: Animalia; phylum: Arthropoda; class:  Insecta; order: Coleoptera; family: Curculionidae; genus: Xylosandrus; specificEpithet: mancus; taxonRank: species; scientificNameAuthorship: (Blandford, 1898); taxonomicStatus: valid; **Location:** country: Thailand; locality: Songkhla Province, Hat Yai District; verbatimElevation: 120 m; verbatimCoordinates: 6°55'20.8"N 100°14'48.8"E; **Identification:** identifiedBy: Sittichaya & Smith; **Event:** eventDate: 12.viii.2024; habitat: tropical rainforest; **Record Level:** type: Non type

##### Distribution

Thailand: Chanthaburi, Chiang Mai, Chumphon, Loei, Nakhon Sri Thammarat, Ranong, Songkhla, Surat Thani.

##### Notes

This species breeds in small branches of *C. inermis* (newly-recorded host). Eggs were laid in 2–3 clusters with different ages with 12–15 progeny (n = 3).

## Discussion

The discovery of 20 xyleborine species from a single fallen *Castanopsis inermis* tree underscores the role of Fagaceae hosts as reservoirs of ambrosia beetle diversity in Southeast Asia. Previous studies have shown that xyleborines are amongst the first colonisers of stressed or fallen trees, where they facilitate fungal colonisation and nutrient recycling (Hulcr and Stelinski 2017, Biedermann and Vega 2020). The high species richness observed here, including twelve new host records, highlights the ecological significance of *C. inermis* as a previously undocumented host for multiple xyleborines. Since *C. inermis* is an ecologically important tree in Asian forests, its colonisation by a diverse bark beetle community suggests that these beetles may contribute to decomposition and nutrient turnover, but also pose potential risks should tree-killing or invasive species expand their host range.

Patterns of host colonisation observed in this study further suggest a partitioning of tree substrates amongst beetle species. Several taxa, such as the members of the genus *Arixyleborus*, *Cyclorhipidion perpilosellum* and *Webbia diversicauda*, were restricted to small branches, whereas *Xyleborus affinis* and *X. perforans* exploited larger trunk and branch diameters, consistent with their biology as strong colonisers of woody tissues (Beaver et al. 2014, Smith et al. 2020). These findings support the view that xyleborines, while often polyphagous, show marked preferences in both host identity and substrate size (Ruzzier et al. 2023). The fact that *C. inermis* represents a new host for many of the species indicates that host specificity amongst xyleborines is more flexible than previously assumed, especially within Fagaceae (Ruzzier et al. 2023). This flexibility has implications for forest health, as shifts in beetle communities may influence the vulnerability of *C. inermis* and related species to pest outbreaks. Documenting these associations thus provides a crucial baseline for biodiversity monitoring and for anticipating potential impacts of ambrosia beetles on forest ecosystems (Poland and Rassati 2018, Gugliuzzo et al. 2021).

## Supplementary Material

XML Treatment for Arixyleborus perbrevis

XML Treatment for Amasa macarthorum

XML Treatment for Amasa versicolor

XML Treatment for Arixyleborus leprosulus

XML Treatment for Arixyleborus malayensis

XML Treatment for Arixyleborus rugosipes

XML Treatment for Arixyleborus suturalis

XML Treatment for Cnestus ater

XML Treatment for Cyclorhipidion perpilosellum

XML Treatment for Debus quadrispinus

XML Treatment for Eccoptopterus limbus

XML Treatment for Euwallacea similis

XML Treatment for Hadrodemius globus

XML Treatment for Leptoxyleborus sordicauda

XML Treatment for Microperus perparvus

XML Treatment for Tricosa cattienensis

XML Treatment for Webbia diversicauda

XML Treatment for Xyleborus affinis

XML Treatment for Xyleborus perforans

XML Treatment for Xylosandrus mancus
